# Mutations in type II Golgi-localized proton pyrophosphatase *AVP2;1/VHP2;1* affect pectic polysaccharide rhamnogalacturonan-II and alter root growth under low boron condition in *Arabidopsis thaliana*

**DOI:** 10.3389/fpls.2023.1255486

**Published:** 2023-08-17

**Authors:** Amarachukwu Faith Onuh, Kyoko Miwa

**Affiliations:** Graduate School of Environmental Science, Hokkaido University, Sapporo, Japan

**Keywords:** *Arabidopsis thaliana*, boron, cell wall, proton pyrophosphatase, rhamnogalacturonan-II (RG-II)

## Abstract

The essential plant nutrient boron is required for the crosslinking of the pectin polysaccharide, rhamnogalacturonan II (RG-II). The synthesis of the pectic polysaccharides takes place in the Golgi apparatus, acidified by proton pumps. AVP2;1/VHP2;1 is a type II proton pyrophosphatase localized in the Golgi apparatus, which possesses proton pumping activity coupled with pyrophosphate hydrolysis. Its activity and expression patterns have been previously revealed but its role in plants remains unknown. The aim of the present work therefore was to explore the physiological role of AVP2;1 in *Arabidopsis thaliana*. In the screening of mutants under low boron, a mutant carrying a missense mutation in *AVP2;1* was isolated. This mutant showed increased primary root growth under low boron conditions but no significant difference under normal boron condition compared to wild type plants. T-DNA insertion caused similar growth, suggesting that reduced function of AVP2;1 was responsible. Root cell observation revealed an increase in meristematic zone length, cell number in meristem and length of matured cell in *avp2;1* mutants compared to wild type under low boron. Calcium concentration was reduced in mutant root cell wall under low boron. RG-II specific sugars also tended to be decreased in mutant root cell wall under low and normal boron conditions. These results suggest that changes in cell wall component by mutations in *AVP2;1* may possibly explain the increased root length of mutants under low boron. This supports the idea that AVP2;1 plays a role in pH homoeostasis in Golgi apparatus for pectin synthesis.

## Introduction

Boron (B) is one of the essential micronutrients of plants. Its deficiency is widespread and various mitigatory approaches such as management of nutrient condition by fertilizer application have been explored. The application of borate fertilizers to make up for the insufficient soil boron is limited by its cost and effectiveness (Güneş et al., 2003; Duran et al., 2018; Fujiyama et al., 2019). Its ineffectiveness is due to the difficulty to ascertain the optimum required fertilizer amount because of the wide range of boron requirement among plant species (Gupta et al., 1985). There is a narrow window between boron deficiency and toxicity and hence inappropriate application of borate fertilizer may lead to toxicity. On the other hand, the use of molecular approach as a mitigatory measure for boron deficiency has been exploited. This involves the overexpression of the boron transporters BOR1, BOR2 and NIP5;1 in plant species like *Brassica napus*, *Zea mays*, *Arabidopsis thaliana*, *Oryza sativa* etc. to improve boron deficiency tolerance (Onuh and Miwa, 2021). Though this approach is useful for the reduction of boron fertilizer application, it still does not completely solve the problem of low-boron stress. This is because upregulating these boron transporters would increase boron uptake thereby leading to a depletion of the limited soil boron in long term. There is therefore a need for a better measure to mitigate low-boron stress.

The primary cell wall is composed of cellulose, hemicellulose, pectin and soluble proteins. Its major component is pectin making up 30-50% of the primary cell wall (Cosgrove and Jarvis, 2012). The pectin component is structurally heterogenous, consisting of three different types of polysaccharide domains: homogalacturonan (HG), rhamnogalacturonan-I (RG-I) and rhamnogalacturonan-II (RG-II) (Mohnen, 2008; Alba and Kontogiorgos, 2017). Minerals such as calcium (Ca) and boron (B) in the pectin, are essential for the crosslinking of HG and RG-II, respectively, which forms the pectin network and aids the overall stability of the primary cell wall. RG-II has HG as a backbone and six distinct side chains A-F (Ndeh et al., 2017; Voxeur et al., 2017) and it has been experimentally demonstrated that RG-II exists as a dimer crosslinked by a borate diester in the plant cell wall (Kobayashi et al., 1996). The major function of boron discovered and experimentally shown so far is its stability of the primary cell wall by crosslinking of RG-II (Matoh et al., 1993; O’Neill et al., 2001).

The pectic polysaccharides of the plant cell wall are synthesized in the Golgi apparatus by the activity of Golgi-localized proteins (Driouich et al., 1993; Lerouxel et al., 2006) and subsequently transported through the TGN (trans-Golgi network) in an acidic condition. With the use of pH sensors, estimated pH was 6.8 and 6.3 in cis-Golgi and TGN in *A. thaliana* protoplasts, respectively (Shen et al., 2013), and it was 6.3 and 5.6 in trans-Golgi and TGN/EE (early endosome) in *A. thaliana* root cells, respectively (Luo et al., 2015). The presence of two types of proton pumps in the Golgi apparatus and TGN which are possibly responsible for its acidification and ion homeostasis has been established in plants. These proton pumps are the vacuolar type ATPase (V-ATPase) (Luo et al., 2015) and proton pyrophosphatase (H^+^-PPase). The H^+^-PPase has a dual function of hydrolysis of pyrophosphate (PPi) into two ortho-phosphates in the cytosol and proton pumping into the Golgi apparatus. The energy generated from the hydrolysis of PPi by this enzyme is what powers its translocation of proton into the Golgi apparatus.

Generally, two types of H^+^-PPase, the type I and type II exist in plants (Drozdowicz et al., 2000). In *A. thaliana*, there are three genes which encode this enzyme. Type I is encoded by *AVP1/VHP1/FUGU5* (At1g15690) and type II by *AVP2;1/VHP2;1* (At1g78920) and *AVP2;2/VHP2;2* (At1g16780). AVP2;1 and AVP1 share 33% amino acid sequence identity whereas AVP2;1 and AVP2;2 share 94% amino acid sequence identity (Segami et al., 2010). The AVP2s, which have a Mg^2+^-binding motif, have been reported to have an obligatory requirement for Mg^2+^ and an insensitivity to potassium ions (K^+^) for their PPi hydrolytic enzymatic activities (Drozdowicz et al., 2000). According to Segami et al. (2010), AVP2s also show characteristics of Zn^2+^ dependency for its enzymatic activity. Segami et al. (2010) have revealed that the AVP1 and the AVP2s whose protein levels are less than 0.3% of AVP1, are localized to the tonoplast (AVP1) and the Golgi apparatus and TGN (AVP2s). So far it has also been found that AVP1 is abundant in shoot apical meristem, leaf primordia and pollen in *A. thaliana* while AVP2s are abundant in young roots, buds, flowers and siliques (Segami et al., 2010; Segami et al., 2014). The functional characterization of AVP1 in *A. thaliana* has been established by studies in mutants and overexpression lines. Some of its known roles are regulation of auxin-mediated organ development and involvement in various abiotic stress tolerance (Li et al., 2005; Kim et al., 2014; Nepal et al., 2020; Zhang et al., 2023). It has also been shown that rather than proton pumping, PPi hydrolysis activity is the major contribution of AVP1 in organ development as demonstrated using *fugu5* mutants (Ferjani et al., 2011; Asaoka et al., 2016). However, little is known about the AVP2s and their mutant phenotype.

In this study, we focused on AVP2;1 and from the screening of *A. thaliana* mutants with enhanced root growth under low boron, a mutant carrying a missense mutation in *AVP2;1* was isolated. T-DNA lines of *AVP2;1* showed similar root growth suggesting that the reduced function of AVP2;1 was responsible for the phenotype. Here we report the phenotypic unveiling of *avp2;1* mutants and the possible effect of *AVP2;1* mutation on cell wall synthesis. These findings give insight into the possible function(s) of AVP2;1 in plants with regards to boron nutrition.

## Materials and methods

### Plant materials and growth conditions

Columbia-0 (Col-0), an ecotype of *Arabidopsis thaliana* (L.) Heynh was used as wildtype (WT) in this study. The mutant number 31A hereafter referred to as *avp2;1-4/vhp2;1-4* used in this study was obtained via the screening of Col-0 seeds mutagenized with ethyl methanesulfonate (EMS) (Hiroguchi et al., 2021). This screening was performed under severe boron deficient (0.03 µM) condition and the mutant showed longer root than Col-0 under this condition and no difference from Col-0 under normal (100 µM) boron condition. This mutant carries a base substitution of guanine to adenine (G800A in CDS and G2492A in full length genomic sequence from the transcriptional start site) which caused an amino acid substitution (G267D in protein). *AVP2;1* (At1g78920) T-DNA insertion mutants SALK_0542912 in Col-0 background (Alonso et al., 2003) and SAIL_165F07 in Col-3 background (Sessions et al., 2002) hereafter referred to as *avp2;1-2/vhp2;1-2* and *avp2;1-3/vhp2;1-3*, respectively, were also used. T-DNA insertion position for both lines is the 6^th^ intron of At1g78920. These T-DNA insertion mutants were obtained from the Arabidopsis Biological Resource Center (ABRC). To determine homozygosity of T-DNA insertion, PCR was performed using the sets of primers listed in **Table S1**.

Seeds were surface sterilized using a washing solution containing 30 mL tap water, 3 mL of bleach (5-10% hypochlorous acid) and 3 drops of detergent, rinsed five times with sterilized ultra-pure water, suspended in ultra-pure water and stored in the dark at 4°C for 4 days before being sown. Plants were cultured in solidified MGRL media (Fujiwara et al., 1992) in which boron concentration was adjusted with boric acid. Growth conditions of 0.06 µM and 0.1 µM boric acid for low boron condition, 0.2 µM, 0.3 µM and 1 µM boric acid for mildly low boron condition, 100 µM boric acid for normal boron condition and 3000 µM boric acid for toxic boron condition were set. The MGRL solid media contained 1% (w/v) sucrose and 1% (w/v) gellan gum (Wako Pure Chemicals). Plants were incubated in a vertical position at 22°C under a 16-hour light/8-hour dark cycle.

### Quantitative reverse-transcription PCR (qRT-PCR)

Surface-sterilized seeds of Col-0 and *avp2;1* mutants were sown in solid medium containing different concentrations of boric acid for 9 days. Roots were harvested and immediately frozen using liquid nitrogen for RNA extraction. The frozen samples were homogenized at 1700 rpm for 15 sec four times using Multi beads shocker (YASUI KIKAI) in a 3 mL tube. Total RNA was extracted using RNeasy Plant Mini Kit (Qiagen, Germany) and treated with RNase-free DNase (Qiagen, Germany) for 30 mins to eliminate genomic DNA contamination. cDNA was synthesized from 0.25 µg total RNA using the PrimeScript RT reagent kit (TAKARA). Expression level of *AVP2;1*, *NIP5;1*, *BOR1*, and *BOR2* was analyzed by qRT-PCR using Thermal Cycle Dice (TAKARA, Japan) with SYBR premix Ex Taq II (TAKARA, Japan). The mRNA level of *AVP2;1* was detected at the 5’ (upstream) portion and 3’ (downstream) portion of T-DNA insertion. *EF1α* was used as a reference gene for normalization of the target mRNA level. Primers used are listed in **Table S1**.

### Measurement of primary root length

For the measurement of primary root length, photographs of 9-day-old seedlings grown under 0.06 µM, 0.1 µM, 0.2 µM, 0.3 µM, 100 µM and 3000 µM boric acid were taken and the digital image was analyzed using an imaging processing software, ImageJ.

### Observation of primary root under stereomicroscopy

Surfaced sterilized seeds of Col-0 and *avp2;1* mutants were grown under 0.1 µM boric acid (low boron), 0.3 µM boric acid (mildly low boron) and 100 µM boric acid (normal boron) in solid media for 5 days. Root tips of plant lines were observed and photographed using a stereomicroscope (OLYMPUS SZX12) equipped with Moticam1080 (Shimadzu).

### Measurement of root cell length and number

Col-0 and *avp2;1-4* mutant were grown under low boron (0.1 µM) and normal boron (100 µM) in MGRL solid media for 5 days. The plant roots were stained with 10 mgL^-1^ propidium iodide (PI: FUJIFILM Wako Pure Chemical, Japan). The imaging data was obtained using a LSM980 (Zeiss, Germany) confocal laser scanning microscope at an excitation and emission wavelength of 543 nm and 543nm-694nm. The obtained images were exported with the Carl Zeiss ZEN 3 blue edition software. The length of fully elongated cortical cells and root apical meristem (RAM) length were measured using ImageJ software. The fully elongated cortical cells or matured cortical cells were defined as the cells within regions of fully developed root hairs (Pacheco-Escobedo et al., 2016). The RAM length was defined as the distance between quiescent center and first elongating cortical cells and the number of cortical cells within the RAM length were counted using the cell counter ImageJ plugin (https://imagej.nih.gov/ij/plugins/cell-counter.html) and regarded as the number of cells in the RAM (Pacheco-Escobedo et al., 2016).

### Dry weight measurement

To obtain root and rosette leaf samples for dry weight measurement, surface sterilized seeds were first grown on rockwool with ultra-pure water and incubated for 7 days at 22°C under a 10-hour light/14-hour dark cycle (short day) and 70% relative humidity. Seedlings were transferred to liquid MGRL media containing 0.15 µM boric acid (low boron), 0.3 µM boric acid (mildly low boron) and 100 µM boric acid (normal boron) and grown for 28 days. Liquid culture was changed weekly during the initial 14 days after transfer, and subsequently changed twice in a week. The rosette leaves and roots of plant lines were harvested and rinsed with ultra-pure water. They were dried at 60°C for 15 days and the dry weight of both rosette leaves and roots was measured.

### Cell wall extraction from root samples

For the analysis of cell wall properties, plants were grown in solid media containing 0.1 µM boric acid (low boron), 0.3 µM boric acid (mildly low boron) and 100 µM boric acid (normal boron) for 9 days and alcohol insoluble residues (AIR) were extracted from their frozen root as described (Matsunaga et al., 2004). About 600-900 root samples were harvested as a single sample for low boron condition and about 300-450, as a single sample for mildly low boron and normal boron conditions. The frozen root tissues were crushed at 1700 rpm for 15 sec in a total of 4 cycles using a multi bead shocker (YASUI KIKAI) and then homogenized with 80% (v/v) ethanol. The homogenates were shaken using a rotator and centrifuged. The insoluble pellets were washed twice with 80% (v/v) ethanol, once with 99.5% (v/v) ethanol, twice with methanol/chloroform (1:1, v/v), once with acetone and twice with ultrapure water. The insoluble residues obtained were freeze-dried using FDU-1200 (EYELA, Japan) for 25 h and treated as cell wall samples.

### Measurement of boron and calcium concentration

To measure total boron and calcium concentration in the rosette leaves and roots, plants were grown in a hydroponic culture system (the same system as dry weight measurement) under 0.1 µM boric acid (low boron), 0.3 µM boric acid (mildly low boron) and 100 µM boric acid (normal boron) and grown for 42 days (7 days in ultra-pure water and 35 days in liquid MGRL media). The concentration for low boron was increased to 0.15 µM boric acid after 19 days of culture in liquid MGRL media due to the severity of boron deficiency. The rosette leaves and roots were then harvested and rinsed with ultrapure water. The harvested samples were dried at 60°C for 9 days (at least more than 3 days) and dry weight was measured. The dried samples were submerged in concentrated HNO_3_ for 3 days at room temperature and first digested at 110°C for about 2 h followed by digestion with H_2_O_2_ for 10 mins. The digested samples were dissolved in 2% HNO_3_. For the measurement of cell wall boron and calcium concentration, about 2 mg of root AIR extracted from 9-d-old plants were treated with HNO_3_ at room temperature for 3 days. The samples were then digested and dissolved in 2% HNO_3_ as described above. Boron and calcium concentrations were measured by inductively coupled plasma mass spectrometry (ELAN DRC-e, PerkinElmer, USA).

### Measurement of RG-II specific sugars

To estimate the amount of RG-II present in root cell wall of plant lines, the measurement of 2-keto-3-deoxy sugars as RG-II specific sugars was performed following a modified thiobarbituric acid protocol described by York et al. (1985). About 1.5 mg of AIR were treated with 5 U of endo-polygalacturnanase from *Pectobacterium carotovorum*, (E-PGALPC, Megazyme, Ireland) in 300 µL of 0.1 M sodium acetate buffer at pH 5.5 for 89 h at 40°C for complete digestion. The enzyme was dialyzed in 0.1 M sodium acetate buffer before use. The suspension was centrifuged three times to remove insoluble residues completely and the supernatant was collected after each centrifuge. To hydrolyze the polysaccharide sugar, 200 µL of the supernatant was mixed with 100 µL of 0.5 M H_2_SO_4_, vortexed and incubated at 100°C for 30 mins. This was followed by cooling down the solution at room temperature for 10 mins, then the addition of 250 µL of 40 mM HIO_4_ dissolved in 62.5 mM H_2_SO_4_, vortexing and incubation at room temperature for 20 mins for a cleavage reaction to generate formylpyruvic acid. 600 µL of 2% Na_2_SO_3_ dissolved in 0.5 M HCl was subsequently added to neutralize excess HIO_4_ and vortexed. A brown coloration was formed upon this addition but quickly disappeared. 500 µL of 25 mM thiobarbituric acid was added, and the mixture was vortexed and incubated at 100°C for 15 mins for pigment generation. 1 mL of DMSO (99.5%) was added to the solution for pigment stabilization and then the mixture was incubated at 24°C for 7 mins. To quantify generated pigments, an absorbance at 548 nm was measured using a spectrophotometer (U-2910, HITACHI High Technologies Corp., Japan). The concentration of 2-keto-3-deoxy sugars were estimated using 2-keto-3-deoxyoctonate ammonium salt (Sigma-Aldrich, USA) as a standard.

### Statistical analysis

Comparisons between the wildtype Col-0 and *avp2;1* mutants were performed using the Dunnett’s test and Student’s t-test.

## Results

### Mutations in *AVP2;1* increased primary root length under low boron

**Figure 1:**
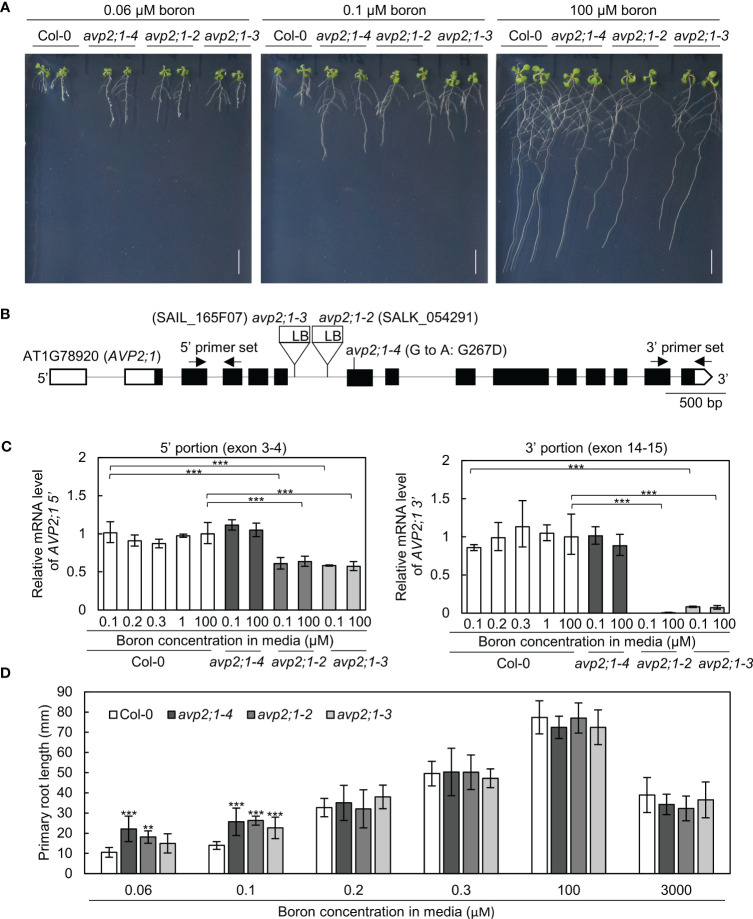
Growth analysis and mRNA level in wildtype Col-0 and *avp2;1* mutants. **(A)** Photos of plants under 0.06 µM (very low), 0.1 µM (low) and 100 µM (normal) boron conditions. (scale bar: 10 mm). **(B)** *AVP2;1* gene structure showing the point mutation, T-DNA insertion positions and primer positions used for mRNA quantification. White and black boxes indicate UTRs and coding regions while the connecting black line indicates the intron. **(C)** mRNA quantification of *AVP2;1* in roots of wildtype Col-0 and *avp2;1* mutants using primers at the 5’ portion and 3’ portion respectively under various concentrations of boron. *EF1α* was used for normalization. Means ± SD are shown (n=3-4). Statistical analysis was not performed for *avp2;1-2* under 0.1 µM at the 3’ portion because it was below detection limit. **(D)** Primary root length of wildtype Col-0 and *avp2;1* mutant lines under various boron concentrations. Means ± SD are shown (n=6-11). All the analysis in **Figure 1** were done using 9-d-old plants grown in solid media. ***P < 0.001,**P < 0.01, compared with wildtype Col-0 under the same boron conditions (Dunnett’s test).

To explore the factors that modulate root growth under boron limitation, *A. thaliana* mutants were screened which showed increased primary root length under low boron supply from EMS-treated Col-0. One of these mutants named number 31A, represented as *avp2;1-4*, showed an enhanced primary root length under a limited supply of boron (0.06 µM and 0.1 µM, **Figures 1A, D**) although the primary root length was not statistically different from Col-0 under normal boron condition (100 µM, **Figures 1A, D**). Through genetic mapping and genomic sequencing, it was revealed that the longer primary root was caused by a single recessive locus and this mutant was found to carry a missense mutation in the 7^th^ exon of *AVP2;1* which encodes a Golgi-localized proton pyrophosphatase (**Figure 1B**). This missense mutation (G800A in CDS) led to an amino acid substitution of glycine to aspartic acid (G267D in protein). G267 is predicted to be located close to the 6th transmembrane domain, and is conserved among AVP1, AVP2;1 and AVP2;2 (Segami et al., 2010; Tojo et al., 2023). To examine if the mutant phenotype was because of its mutation in *AVP2;1*, two T-DNA lines of *AVP2;1* were obtained. These two lines represented as *avp2;1-2* and *avp2;1-3* respectively, carry a T-DNA insertion in the 6^th^ intron of *AVP2;1* (**Figure 1B**). Both T-DNA lines showed increased primary root under low boron but no differences under normal boron supply similar to *avp2;1-4* (**Figures 1A, D**), supporting that the mutations in *AVP2;1* were responsible for the increased primary root length under low boron conditions.

To check *AVP2;1* mRNA expression in the mutants, *AVP2;1* mRNA was quantified by qRT-PCR using two sets of primers to detect the upstream portion (5’, exon 3-4) and downstream (3’, exon 14-15) of T-DNA insertion. In Col-0, with the use of both sets of primers, *AVP2;1* mRNA level was not obviously changed under various boron concentrations (**Figure 1C**) suggesting that the mRNA expression of *AVP2;1* in roots is not dependent on boron nutrition. Under low (0.1 µM) and normal (100 µM) boron, *AVP2;1* mRNA was not changed in *avp2;1-4* when compared to Col-0 using both upstream and downstream primers. However, in *avp2;1-2* and *avp2;1-3*, *AVP2;1* mRNA was reduced to 60% in the upstream portion of the T-DNA insertion. In the downstream portion of insertion, *AVP2;1* mRNA level was reduced to 0.5% and 7-10% in *avp2;1-2* and *avp2;1-3*, respectively under both boron conditions compared to Col-0 (**Figure 1C**). This suggests that although the destabilized 5’ incomplete portion of mRNA was likely expressed, *AVP2;1* mRNA is not at least overexpressed in T-DNA insertion lines. This shows that the T-DNA insertion mutants, *avp2;1-2* and *avp2;1-3*, are knockdown mutants suggesting that reduced function of AVP2;1 is responsible for the mutant phenotype.

To examine the growth response of the *avp2;1* mutants to different boron conditions, plants were grown under various range of boron concentration. *avp2;1* mutants exhibited longer primary roots only under low boron conditions (0.06 and 0.1 µM) compared to Col-0 but showed no significant differences under mildly low (0.2 and 0.3 µM), normal (100 µM) and even toxic (3000 µM) boron conditions (**Figure 1D**).

To check the possibility of the involvement of *AVP2;1* in root growth under other stress conditions, mutant lines were grown under low phosphorus and low pH, respectively. No differences were found between the *avp2;1* mutants and Col-0 under these conditions (**Figures S1A, B**). Taken together, these results indicate that the reduced expression or function of *AVP2;1* causes an increase in the primary root length under a limited supply of boron and this increase is likely observed specifically under low boron.

### Alleviated inhibition of both cell division and elongation was observed in *avp2;1* mutants

**Figure 2:**
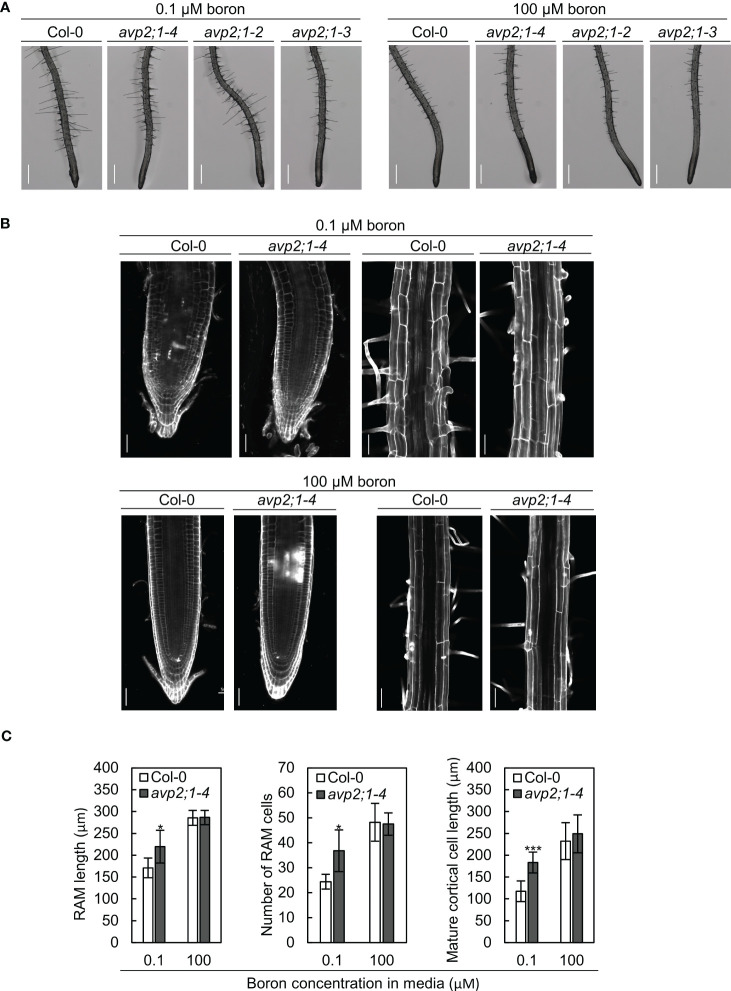
Observation of the root cells of Col-0 and *avp2;1* mutants. **(A)** Stereomicroscopic images of root tips of wildtype Col-0 and *avp2;1* mutants grown under low (0.1 µM) and normal (100 µM) boron conditions. (scale bar: 400 µm). **(B)** Confocal images of roots of wildtype Col-0 and *avp2;1-4* grown under low (0.1 µM) and normal (100 µM) boron conditions showing its meristematic zone and mature zone. (scale bar: 50 µm). **(C)** Root apical meristem (RAM) length (n=5-7 plants), number of RAM cells (n=5-7 plants), and mature cortical cell length (n=5-7 plants, means of 2-12 cells per plant were taken) of wildtype Col-0 and *avp2;1-4* under 0.1 µM and 100 µM boron conditions. Means ± SD are shown. All the analysis in **Figure 2** were done using 5-d-old plants grown in solid media. ***P < 0.001,*P < 0.05, compared with wildtype Col-0 under the same boron conditions (Student’s t-test).

To characterize the effects of *avp2;1* mutations on root cells, the root tips of 5-d-old plants grown under low (0.1 µM), mildly low (0.3 µM), and normal (100 µM) boron were first observed under a stereomicroscope. Although primary root length was not obviously different between Col-0 and *avp2;1* mutants at this age under all the boron conditions (**Figure S2A**), longer meristematic and elongation zones were observed in *avp2;1* mutants compared to Col-0 under low (0.1 µM) boron condition (**Figure 2A**) and no difference was observed under mildly low (0.3 µM) boron (**Figure S2B**) and normal (100 µM) boron condition (**Figure 2B**). There were also no distinguishing differences in the root hair distribution between wildtype Col-0 and *avp2;1* mutant lines under both low and normal boron conditions. The root cells of Col-0 and *avp2;1-4* mutant was further examined using a confocal microscope in 5-d-old plants. Under low (0.1 µM) boron, the shape of the root cells of Col-0 were collapsed and the root cell arrangement was damaged; however, *avp2;1-4* root cells showed a mitigated inhibition in cell shape and cell arrangement (**Figure 2B**). In addition, an increase in meristematic zone length, cell number in root apical meristem (RAM), and length of matured cortical cell was observed in *avp2;1-4* compared to Col-0 under low boron condition, and no differences was found under normal (100 µM) boron condition (**Figure 2C**). These observations suggest that inhibition of both root cell elongation and division caused by low boron was alleviated by *avp2;1* mutation accounting for the enhanced primary root growth.

### Boron concentration in roots and rosette leaves was not different between Col-0 and *avp2;1* mutants

**Figure 3:**
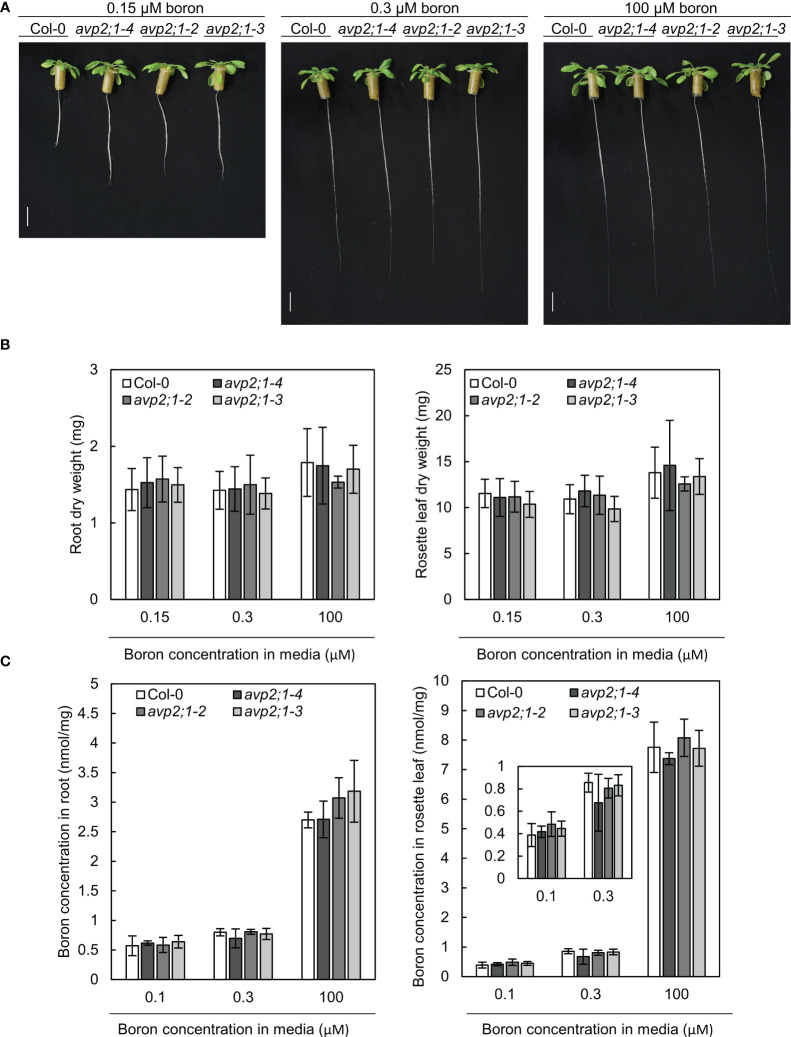
Growth in hydroponic culture and boron concentration in root and rosette leaves. **(A)** Representatives of wildtype Col-0 and *avp2;1* mutants grown in a hydroponic culture under low (0.15 µM), mildly low (0.3 µM) and normal (100 µM) boron conditions for 35 days. (scale bar: 20 mm). **(B)** Dry weight of wildtype Col-0 and mutant roots and rosette leaves grown under the same condition as **(A)**. **(C)** Boron concentration in root and rosette leaves of wildtype Col-0 and *avp2;1* mutants grown for 42 days under low (0.1 µM, the B concentration in media was increased to 0.15 µM after 19 days of culture in liquid MGRL media), mildly low (0.3 µM) and normal (100 µM) boron conditions using hydroponic culture. Means ± SD are shown (n=5-6). *P* > 0.05, compared with wildtype Col-0 under the same boron conditions (Dunnett’s test).

To explore the mechanisms behind the increased root length of *avp2;1* mutants under limited supply of boron, the possibility of an enhancement in boron uptake or transport in mutant lines was considered. To measure tissue boron concentration, plants were hydroponically grown so that plant roots can freely access the media. In hydroponic culture, *avp2;1* mutants also exhibited longer root length compared to the wildtype Col-0 under low (0.15 µM) boron condition when Col-0 root elongation was inhibited. No obvious difference in root length was found among plant lines under mildly low (0.3 µM) and normal (100 µM) boron conditions (**Figure 3A**). This shows the consistency of the mutant phenotype irrespective of the plant culture system. In both root and rosette leaf dry weight, no significant differences were observed between Col-0 and *avp2;1* mutants (**Figure 3B**). In the concentration of total boron in roots and rosette leaves, no differences were observed between the wildtype Col-0 and *avp2;1* mutant lines irrespective of the boron concentration in the media (**Figure 3C**). This suggests that the mutations in *AVP2;1* did not primarily affect the boron transport.

**Figure 4:**
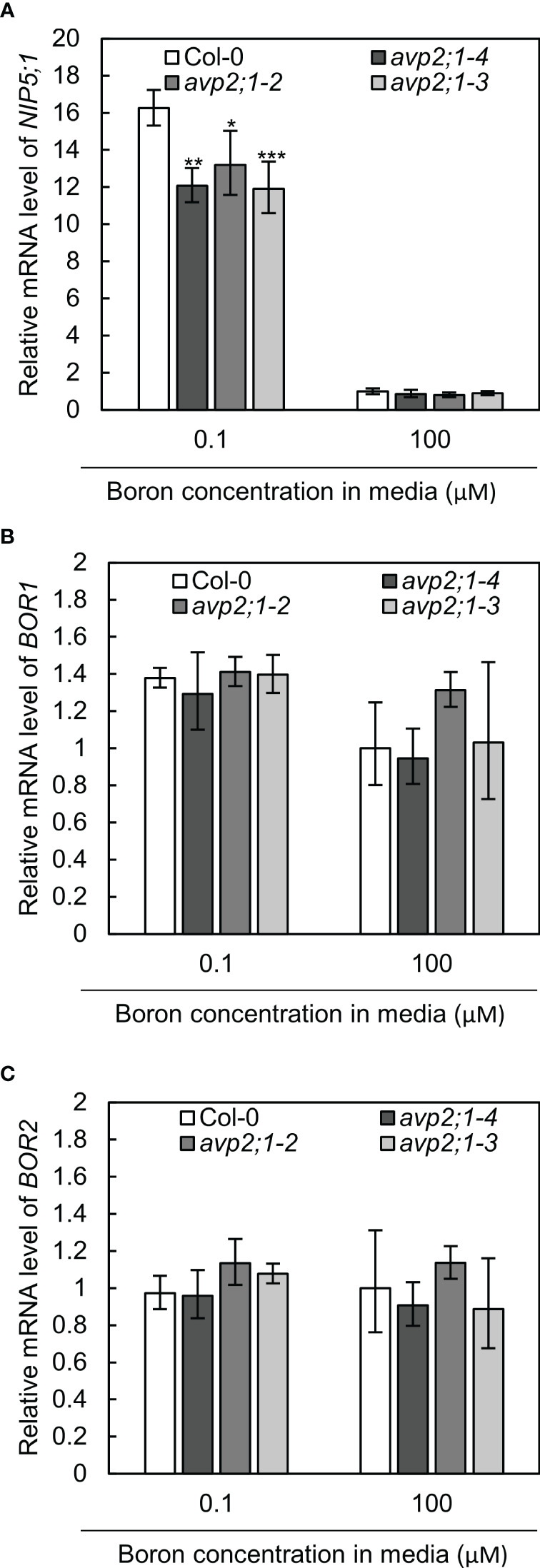
mRNA levels of boron transporter genes. **(A–C)** Relative mRNA quantification of *NIP5;1* **(A)** *BOR1* **(B)** and *BOR2* **(C)** in root of wildtype Col-0 and *avp2;1* mutant lines under low (0.1 µM) and normal (100 µM) boron condition. mRNA extracted from 9-d-old plants grown in solid media were used for all analysis. *EF1α* was used for normalization. The data was standardized by the value of Col-0 under normal (100 µM) boron condition which was set as 1. Means ± SD are shown (n=3-4). ***P < 0.001, **P < 0.01, *P < 0.05 compared with wildtype Col-0 under the same boron conditions (Dunnett’s test).

To further examine the effects of *avp2;1* mutation in boron transport, the mRNA expression of *NIP5;1*, *BOR1* and *BOR2* were checked in wildtype Col-0 and *avp2;1* mutant lines under low (0.1 µM) and normal (100 µM) boron conditions. *NIP5;1*, *BOR1* and *BOR2* encode major boron transporters required for root growth under low boron (Onuh and Miwa, 2021). No increase was detected in the mRNAs of these transporter genes in the mutant lines under low and normal boron conditions (**Figures 4A–C**). *NIP5;1* mRNA is induced by low boron (Tanaka et al., 2011). *NIP5;1* mRNA levels in all the three *avp2;1* mutants were rather reduced to 73-74% of that of Col-0 under low boron (**Figure 4A**). This decrease in *NIP5;1* mRNA in roots could be an indirect consequence of the increased root length in *avp2;1* mutants under low boron (**Figure 1A**). *NIP5;1* promoter activity is more strongly detected in the elongation zone compared to the mature portion of the roots (Takano et al., 2006). Compared to Col-0 in which root elongation was severely inhibited (**Figure 1A**), the relative proportion of the elongation zone to the entire root could be smaller in *avp2;1* mutants, resulting in the reduction in relative level of *NIP5;1* mRNA under low boron.

Taken together, these results suggest that the upregulation of boron transport was not likely the cause of the enhanced root length exhibited by the *avp2;1* mutant lines under low boron.

### Reduced calcium and RG-II specific sugars were observed in root cell wall of *avp2;1* mutants

**Figure 5:**
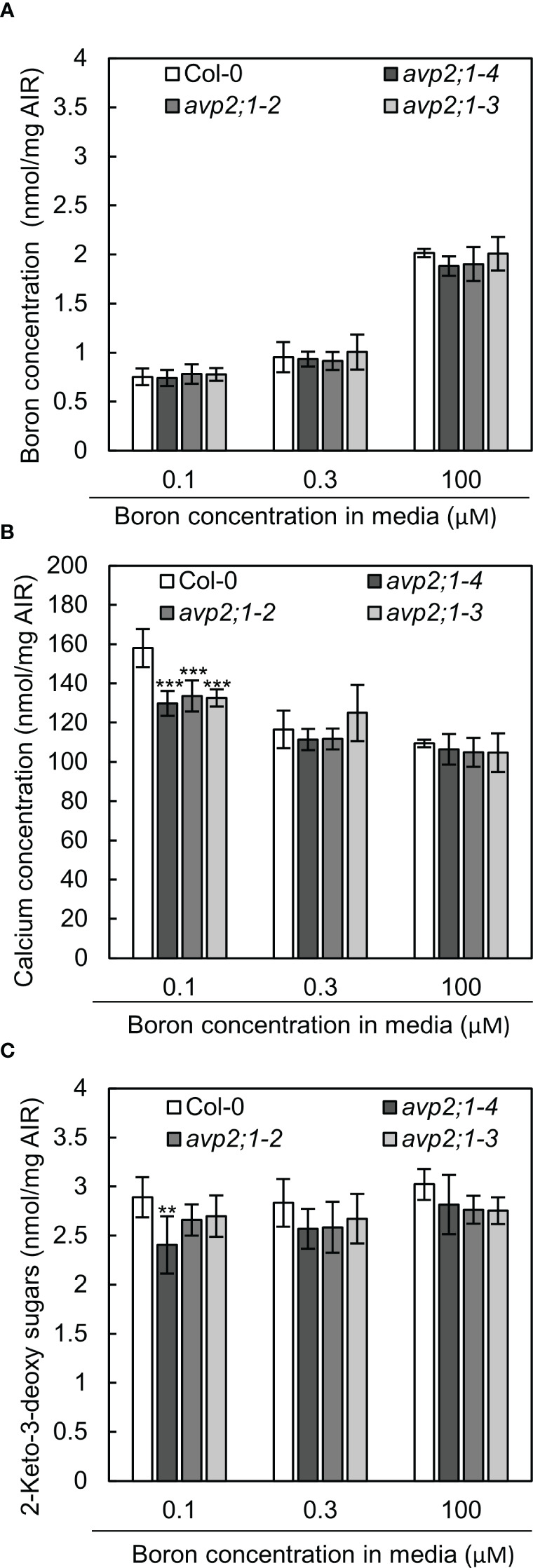
Boron, calcium and RG-II specific sugars in root cell wall extracted as alcohol insoluble residue (AIR). **(A-C)** Concentration of boron **(A)** calcium **(B)** and RG-II specific sugar, 2-keto-3-deoxy sugars **(C)** in root AIR of wildtype Col-0 and *avp2;1* mutants. The same AIR extracted from 9-d-old plants grown under low (0.1 µM), mildly low (0.3 µM) and normal (100 µM) boron conditions in solid media were used for all analysis. Means ± SD are shown (n=4-6). ***P < 0.001, **P < 0.01, compared with wildtype Col-0 under the same boron conditions (Dunnett’s test).

Due to the role of boron in plant cell wall stability by crosslinking of two monomers of RG-II, changes in cell wall components were considered as a possible mechanism of the enhanced root growth of *avp2;1* mutants under a low boron supply. To explore this possibility, concentrations of the cell wall minerals, calcium, and boron, were measured in root cell wall under low (0.1 µM), mildly low (0.3 µM), and normal (100 µM) boron conditions. No difference was found in boron concentration in cell wall under low and mildly low boron condition, but *avp2;1* mutant lines showed a slight tendency of reduction compared to the wildtype Col-0 under normal boron conditions (**Figure 5A**). Since boron binding to RG-II is assumed to be saturated when sufficient boron is supplied, this suggests a possibility of a reduced boron binding capacity in *avp2;1* mutant cell wall. On the other hand, the concentration of calcium in the cell wall was generally increased in all the plant lines under low boron compared to mildly low and normal boron (**Figure 5B**), probably as a compensation of the reduced borate crosslinking in the cell wall. Under low boron, *avp2;1* mutant lines showed a significant decrease by15-18% in cell wall calcium concentration (**Figure 5B**), but there were no differences under mildly low and normal boron conditions.

To further confirm the possibility of a reduced boron binding capacity in *avp2;1* mutant root cell wall, RG-II amount was estimated by measuring 2-keto-3-deoxy sugars which are RG-II specific sugars. There was a 6-17% decrease of RG-II specific sugars in *avp2;1* mutant lines compared to wildtype Col-0 under all the three boron conditions although a statistical difference was found only in *avp2;1-4* under low B (**Figure 5C**).

These results support that a*vp2;1* mutations did affect cell wall components and possibly cell wall stability. This change could probably explain the increased root growth of *avp2;1* mutants under a limited boron supply.

## Discussion

Previous studies have successfully identified and characterized the *A. thaliana* type II H^+^-PPase, AVP2;1 as a proton pump localized to the Golgi apparatus and TGN (Segami et al., 2010). However, its physiological role and their mutant phenotype remains poorly understood in plants. In this study, we characterized *avp2;1* mutants from the view of boron deficiency stress and found that mutations in *AVP2;1* affected plant growth under low boron conditions and cell wall components under low and normal boron conditions. To describe the mechanism behind this, we propose that a reduction of AVP2;1 decreases pH pumping activity in Golgi apparatus and this possibly leads to the changes in biological processes in Golgi apparatus such as reduction in pectin synthesis, which causes reduced sensitivity to low boron by a reduced boron requirement.

### H^+^ pumping rather than PPi hydrolysis is likely a key for *avp2;1* mutant root growth

AVP2;1 like other H^+^ -PPases has the dual role of PPi hydrolysis and proton pumping. To have a holistic picture of the functioning of AVP2;1 in plants, it is important to understand which role of AVP2;1 causes the observed mutant phenotypes. In the case of AVP1/VHP1, *fugu5* mutant was used to demonstrate this. The *fugu5-1* is a loss-of-function *AVP1* mutant which has a null PPase activity resulting in reduced shoot growth, impaired size and shape of cotyledon, and reduced hypocotyl length of etiolated seedlings (Ferjani et al., 2011; Asaoka et al., 2016). The introduction of cytosolic soluble inorganic pyrophosphatase (IPP) of *S. cerevisiae* under the control of the *AVP1* promoter into the *fugu5-1* mutant, was able to rescue these phenotypes (Ferjani et al., 2011). This suggested that rather than proton pumping, hydrolysis of cytosolic PPi is the major physiological role of AVP1. This was further confirmed using an uncoupling mutated variant of AVP1 that could hydrolyze PPi but had no proton pumping activity. The introduction of this mutant variant into *fugu5-3* rescued shoot growth as when native H^+^-PPase (AVP1) was introduced (Asaoka et al., 2016), thereby supporting that the hydrolytic role rather than proton pumping of AVP1, is essential for plant growth. On the other hand, Tojo et al. (2023) has revealed the negligible contribution of PPi hydrolytic role of AVP2;1 in growth of *A. thaliana* seedling. This was done by phenotypic characterization of the *AVP2;1* mutant, *vhp2;1-1*/*avp2;1-1* (SAIL_512_B02) and *fugu5-1vhp2;1-1* double mutant. Phenotypes related to loss of PPase activity such as alterations in cotyledon shape and hypocotyl elongation defects in etiolated seedlings were not observed in *vhp2;1-1* and neither was any additional effect observed in *fugu5-1vhp2;1* compared to *fugu5-1*. We also tested the response of *avp1-4* (Yang et al., 2018) to low boron stress. However, no difference from Col-0 was observed (**Figure S3**) in contrast to *avp2;1* mutants. Considering the minimal contribution of AVP2;1 in PPi hydrolysis for the growth of *A. thaliana* and the plant growth observed in *avp1-4*, we suggest that rather than the hydrolysis of cytosolic PPi, proton pumping into the Golgi apparatus is the primary component driving the *avp2;1* mutant root growth under low boron conditions.

### Change of H^+^ pumping activity in the Golgi apparatus possibly affects pectin synthesis including RG-II

**Figure 6:**
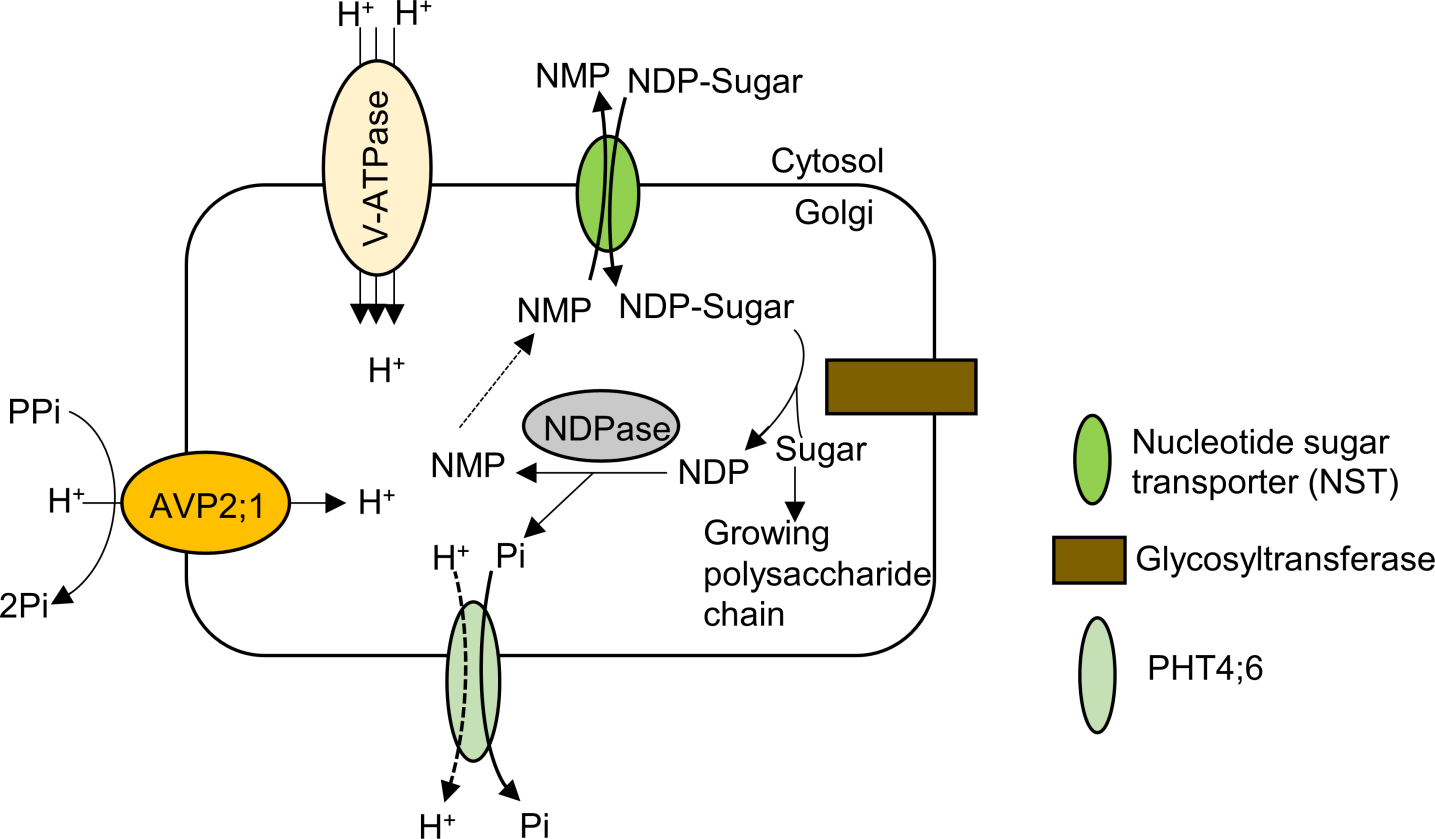
Model of pectin synthesis and the contribution of AVP2;1 to the maintenance of ion homeostasis in the Golgi apparatus. By the activity of the nucleotide sugar transporter (NST) which is an NDP-sugar/NMP antiporter, nucleoside di-phosphate sugar (NDP-sugar) is imported from the cytosol into the Golgi lumen. The sugar moiety is then transferred to a growing chain of polysaccharide via the action of glycosyltransferase and the resulting NDP is hydrolyzed by NDPase to nucleoside monophosphate (NMP) and inorganic phosphate. The NMP concentration gradient drives transport of NDP-sugar via NSTs as NDP-sugar/NMP antiporters. The inorganic phosphate released from NDP hydrolysis is exported into the cytosol from Golgi apparatus via a phosphate transporter (PHT4;6). AVP2;1 helps to maintain ionic homeostasis and acidic state of the Golgi apparatus via its proton pumping activity thereby enhancing the process of pectic polysaccharide synthesis.

The Golgi apparatus is involved in sorting and transporting proteins to cellular compartments as well as assembling and exporting the non-cellulosic polysaccharides of the cell wall matrix including pectin in plants (Driouich et al., 2012). For non-cellulosic cell wall polysaccharide synthesis in Golgi apparatus, by the activity of the nucleotide sugar transporter (NST), nucleoside di-phosphate sugar (NDP-sugar) is imported from the cytosol into the Golgi lumen, supplying substrate for glycosyltransferase (**Figure 6**). The sugar moiety is then transferred to a building chain of polysaccharide via the action of glycosyltransferase and the resulting NDP is hydrolyzed by NDPase to nucleoside monophosphate (NMP) and inorganic phosphate. The NMP concentration gradient drives transport of NDP-sugar via NSTs as NDP-sugar/NMP antiporters. The inorganic phosphate released from NDP hydrolysis is exported into the cytosol from Golgi apparatus via a phosphate transporter (Reyes and Orellana, 2008; **Figure 6**).

Assuming H^+^ pumping activity of AVP2;1 as its key contribution to plant growth, we propose that a reduced function of AVP2;1 would increase pH and affect ion homeostasis in the Golgi apparatus. The effect of these changes would likely affect biochemical processes in the Golgi apparatus including pectin synthesis (**Figure 6**) and in this study, we have observed a tendency of a reduction in RG-II amount under various boron concentrations in *avp2;1* mutant cell wall (**Figure 5**). This proposition is in line with Reyes and Orellana (2008) hypothesis that the activity of glycosyltransferase could be affected by ion homeostasis and pH. pH dependent activity of some *A*. *thaliana* glycosyltransferases such as RG-I: galacturonsyltransferase (RGGAT1) (Amos et al., 2023), HG: galacturonosyltransferase (GAUT1:GAUT7) (Amos et al., 2018), RG-I: rhamnosyltransferase (RRT1) (Takenaka et al., 2018), RG-II: xylosyltransferase (RGXT1) (Petersen et al., 2009) involved in pectin synthesis has been reported. These *in vitro* analyses showed an optimal pH for these glycosyltransferases to be pH 6.5 (RGGAT1), pH 7.2 (GAUT1:GAUT7), pH 7 (RRT1) and around pH 7 (RGXT1). However, this may be different from the actual activity within the cell considering the pH range of the Golgi apparatus. In addition, it has been shown in human cancer cells that a slight increase in the Golgi pH affects the formation of glycosyltransferase complexes and also induces mislocalization of glycosyltransferase from the Golgi apparatus into endosomal compartments (Kellokumpu, 2019).

The Golgi-localized inorganic phosphate transporter, PHT4;6 in *A. thaliana* has been identified to release inorganic phosphate (Pi) into the cytosol from the Golgi lumen (Guo et al., 2007). Although it has not been experimentally demonstrated, it is hypothesized that the release of the lumenal Pi by PHT4;6 is dependent on proton gradients (Guo et al., 2007). The lumenal Pi if not released, could hamper activities in the Golgi apparatus including pectin synthesis.

Considering the localization of AVP2;1 to the TGN, there is also a possibility that the pectin trafficking is hampered/affected by a reduced function of AVP2;1. This probably could lead to a reduced amount of pectin which is trafficked or deposited to the cell wall.

We also observed the reduction of calcium concentration in cell wall of *avp2;1* mutants under low boron compared to wildtype Col-0 (**Figure 5**). The HG is crosslinked by calcium and hence, a reduction in cell wall calcium concentration could imply a reduced HG. We propose that in addition to RG-II polysaccharide, HG polysaccharide could be also affected by changes in the state of the Golgi apparatus caused by the reduced H^+^ pumping activity of AVP2;1. It is also possible that the reduction of calcium-crosslinked HG is due to a secondary effect induced by growth change in mutants rather than a direct effect of reduction in H^+^ pumping activity of AVP2;1. Pectin methylesterase (PME) catalyzes the demethylesterification of HG thereby increasing calcium crosslinking. It has been found that the activity of PME is induced by boron deficiency (Yan et al., 2021) possibly for compensation of borate-RG-II crosslinking. Hence, due to relieved severity of boron deficiency in *avp2;1* mutants under low boron supply, the demethylesterification activity of the PME could be reduced resulting in the decrease of cell wall calcium concentration.

### Decrease in RG-II amount may possibly reduce boron requirement in *avp2;1* mutants

One of the major effects of boron deficiency on plants is the damage of growing points, which include inhibition of root cell elongation and division leading to a cessation of root elongation (Dell and Huang, 1997). However, *avp2;1* mutant lines showed less sensitivity to this (**Figure 2**). Following the observed tendency of reduced amount of RG-II by 6-17% in these mutants (**Figure 5**), we suggest that this reduced sensitivity to low boron is because of a reduction in boron requirement. Reduced boron requirement means that a smaller amount of boron can satisfy the boron demand possibly due to the reduced boron binding sites for RG-II in *avp2;1* mutants. In line with our study, Hiroguchi et al. (2021) has also shown that 20-30% reduction in RG-II enhances root elongation under limited supply of boron. In this case, increased root meristem was not clearly observed under low boron condition in *tmn1* mutants at 4 days compared to this current study of *avp2;1* mutants at 5 days (**Figure 2**). However, a tendency of an increased root meristem was observed in the *tmn1* mutant still at 4 days and at a longer incubation time under low boron condition in hydroponic culture (Hiroguchi et al., 2021).

It has also been reported that there is a positive correlation between pectin amount and cell wall boron concentration which may represent amount of boron required by plants for tissue development among different plant species (Hu et al., 1996). More directly, a positive correlation between cell wall boron concentration and RG-II amount has been revealed (Matoh et al., 1996). This implies pectin amount or RG-II amount determines sensitivity to boron deficiency hence the reduced sensitivity to low boron in *avp2;1* mutants. We suggest that cell wall modification would be another approach for conferring low-boron tolerance.

### The contribution of AVP2;1 to pH homeostasis in Golgi apparatus may be minimal

In our study, *avp2;1* mutants showed a significant increase in primary root growth accompanied by increased root cell division and elongation when compared to wildtype Col-0 under low boron. This increment of growth was however not observed in the leaves of *avp2;1* mutants (**Figures 1**, **3**) possibly because AVP2s protein is predominately expressed in roots but not in young and mature leaves (Segami et al., 2010). Again, differences between the wildtype Col-0 and *avp2;1* mutants were not observed under normal boron condition. One possible explanation to this could be a minor contribution of AVP2;1 to pH homeostasis in Golgi apparatus because it is assumed that V-ATPase is the major proton pump in the Golgi apparatus. Result of the analysis of RG-II specific sugars revealed a 6-7% consistent reduction in *avp2;1* mutants compared to wildtype Col-0 under both low and normal boron condition except for *avp2;1-4* which showed 17% reduction under low boron condition. Under normal boron conditions, most of RG-II is crosslinked by sufficient supply of boron therefore, a 6-7% reduction of RG-II may not greatly affect the plant growth. However, under low boron conditions, when borate crosslinking of RG-II is likely a major determinant for cell division and elongation, this slight change of RG-II could affect the plant growth.

AVP2;2 is an isoform of AVP2;1 and shares about 94% amino acid sequence identity (Segami et al., 2010). Based on the amino acid sequence identity of the two proteins, we examined *avp2;2* mutants to check if AVP2;2 has a similar role in low boron as AVP2;1. The analysis of the growth of *avp2;2* mutants, however, revealed that unlike *avp2;1* mutants, *avp2;2* mutants showed no significant differences from Col-0 under low and normal boron conditions (**Figure S4**). This could be because of its very low transcript amount compared to *AVP2;1* hence no noticeable change in *avp2;2* mutants. Another possibility is that though AVP2;1 and AVP2;2 share great similarities, their functions do not overlap, and reduction of low boron sensitivity may be specific to AVP2;1.

In conclusion, this study supports an idea that AVP2;1 plays a role in proton pumping and acidification of Golgi apparatus for maintenance of pectin synthesis by examination of *avp2;1* mutants. It also proposes the reduction of boron requirement by molecular approach as a sustainable strategy against low boron stress.

## Data availability statement

The original contributions presented in the study are included in the article/**Supplementary Material**. Further inquiries can be directed to the corresponding author.

## Author contributions

AO: Conceptualization, Data curation, Formal Analysis, Funding acquisition, Investigation, Methodology, Resources, Validation, Writing – review & editing, Writing – original draft. KM: Conceptualization, Data curation, Formal Analysis, Funding acquisition, Investigation, Methodology, Resources, Validation, Writing – review & editing, Project administration, Supervision.
