# Supplementary material for: Mutations in type II Golgi-localized proton pyrophosphatase AVP2;1/VHP2;1 affect pectic polysaccharide rhamnogalacturonan-II and alter root growth under low boron condition in Arabidopsis thaliana
Source: Front Plant Sci. 2023 Aug 17;14:1255486. doi: 10.3389/fpls.2023.1255486 (PMC10469939; doi:10.3389/fpls.2023.1255486)
Supplement: Supplementary file 1 [file DataSheet_1.pdf]

Supplementary Material

Mutations in type II Golgi-localized proton pyrophosphatase *AVP2;1/VHP2;1* affect pectic polysaccharide rhamnogalacturonan-II and alter root growth under low boron condition in *Arabidopsis thaliana*

Amarachukwu Faith Onuh, Kyoko Miwa*

*** Correspondence:** Kyoko Miwa: miwakyoko@ees.hokudai.ac.jp

Supplementary materials and method

Plant materials and growth condition under other stresses

Surface sterilized wildtype Col-0 and *avp2;1* mutants were used. For low phosphorus treatment, plants were grown in half strength Murashige and Skoog (MS) solidified media containing 3.5 mM MES and pH adjusted to 5.68 using KOH. The phosphorus concentration was adjusted with KH_2PO_4. Growth conditions of 12.5 µM phosphate for low phosphorus condition and 625 µM phosphate for normal phosphorus condition were set. The MS media also contained 1% (w/v) sucrose and 1% (w/v) agar (for plant culture medium, Wako Pure Chemicals with catalogue number of 010-15815). For the pH test, half strength of MS solidified media solution was prepared containing 3.5 mM MES. pH was set as 4.29 (low pH) with HCl and as 5.7 (normal pH) with KOH. The solid media also contained 1% (w/v) sucrose and 1% (w/v) agar (for plant culture medium, Wako Pure Chemicals with catalogue number of 010-15815). Plants were incubated in vertical positions at 22°C under a 16-hour light/8-hour dark cycle until analysis.

Genotyping of T-DNA insertion mutants of *avp1* and *avp2;2* mutants

To determine homozygosity of T-DNA insertion mutants, DNA was extracted from shoots of 7-d-old T-DNA lines (*avp1* and *avp2;2*) and wildtype Col-0 seedlings grown under 100 µM boron condition. PCR was performed on *avp1* mutant GABI_596C07 (Yang *et al.*, 2018) represented as *avp1-4* and *avp2;2* mutants SALK_044701 (*avp2;2-1*) and SALK_138132 (*avp2;2-2*) (Tojo *et al.*, 2023) using the primers listed in Table S1.

Supplementary Table S1. Primers used in this study

Stock no	Sequence (5’>3’)	Purpose	Product size
**5**<br>**654**	ATTTTGCCGATTTCGGAAC<br>ATGCCCCAAACAAGGATGTCA	Genotyping of *avp2;1-2*<br>SALK_0542912 (T-DNA LB)	~500 bp
**653**<br>**654**	GGCTGTGATTGGTATCGCCA<br>ATGCCCCAAACAAGGATGTCA	Genotyping of *avp2;1-2*<br>SALK_0542912 (AT genome)	658 bp
**6**<br>**654**	TTCATAACCAATCTCGATACAC<br>ATGCCCCAAACAAGGATGTCA	Genotyping of *avp2;1-3*<br>SAIL_165F07 (T-DNA LB)	~513 bp
**653**<br>**654**	GGCTGTGATTGGTATCGCCA<br>ATGCCCCAAACAAGGATGTCA	Genotyping of *avp2;1-3*<br>SAIL_165F07 (AT genome)	658 bp
**1201**<br>**1202**	CATCATCGTGTTCGTCTTCAC<br>GAATCACAAAAGCTAGCAAAATG	*AVP2;1* mRNA quantification at 5’ portion	200 bp
**1203**<br>**844**	ATACATAGAGACCGGGGCACTT<br>AAGCCGGGATGGATAAGTAACG	*AVP2;1* mRNA quantification at 3’ portion	199 bp
**226**<br>**227**	AATCTCGCAGCGGAAACG<br>TGGAGTCGAACTTGAACTTGTC	*BOR1* mRNA quantification	141 bp
**1199**<br>**1200**	CATCTCGCAGTACCGGAAGCT<br>AGCCTTGGACTCATCTCACCT	*BOR2* mRNA quantification	218 bp
**139**<br>**140**	CACCGATTTTCCCTCTCCTGAT<br>GCATGCAGCGTTACCGATTA	*NIP5;1* mRNA quantification	151 bp
**61**<br>**62**	CCTTGGTGTCAAGCAGATGA<br>GAAGACACCTCCTTGATGATTT	*EF1α* mRNA quantification	102 bp
**1049**<br>**259**	ATTGCTTTCGTGCTTGGTGC<br>ATATTGACCATCATACTCATTGC	Genotyping of *avp1-4*<br>GABI_596C07 (T-DNA LB)	626 bp
**1049**<br>**1050**	ATTGCTTTCGTGCTTGGTGC<br>AATGGGTAGCACATGGCAGT	Genotyping of *avp1-4*<br>GABI_596C07 (AT genome)	641 bp
**5**<br>**765**	ATTTTGCCGATTTCGGAAC<br>CAGAGAAAGATAGGAGCCATGA	Genotyping of *avp2;2-1*<br>SALK_044701 (T-DNA LB)	~289 bp
**764**<br>**765**	GGCTATAATATCACCCATTGCAG<br>CAGAGAAAGATAGGAGCCATGA	Genotyping of *avp2;2-1*<br>SALK_044701 (AT genome)	560 bp
**991**<br>**5**	TGAGCCAGCAGGTATGAGTC<br>ATTTTGCCGATTTCGGAAC	Genotyping of *avp2;2-2*<br>SALK_138132 (T-DNA LB)	~650 bp
**991**<br>**992**	TGAGCCAGCAGGTATGAGTC<br>CGCAAACATTAGCATTGCAGC	Genotyping of *avp2;2-2*<br>SALK_138132 (AT genome)	866 bp

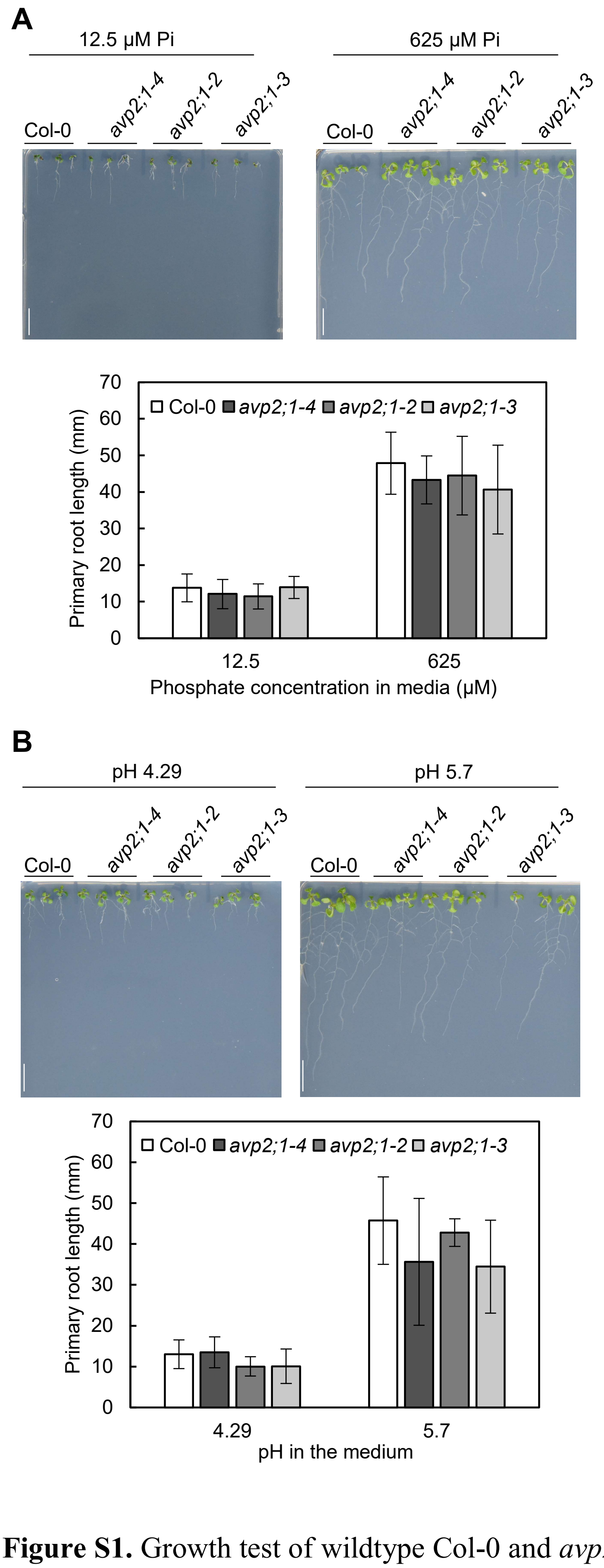

Supplementary Figure S1. Growth test of wildtype Col-0 and *avp2;1* mutants under low phosphorus and low pH conditions. **(A), (B)** Primary root of wildtype Col-0 and *avp2;1* mutants under low (12.5 μM) and normal (625 μM) phosphate conditions (A) and low pH (4.29) and normal pH (5.7) conditions (B). (Scale bar: 10 mm). All growth test was performed using 9-d-old plants grown in solid media. Mean ±SDs are shown (n=13-27). $P > 0.05$ compared with wildtype Col-0 under the same phosphorus and pH conditions (Dunnett's test)

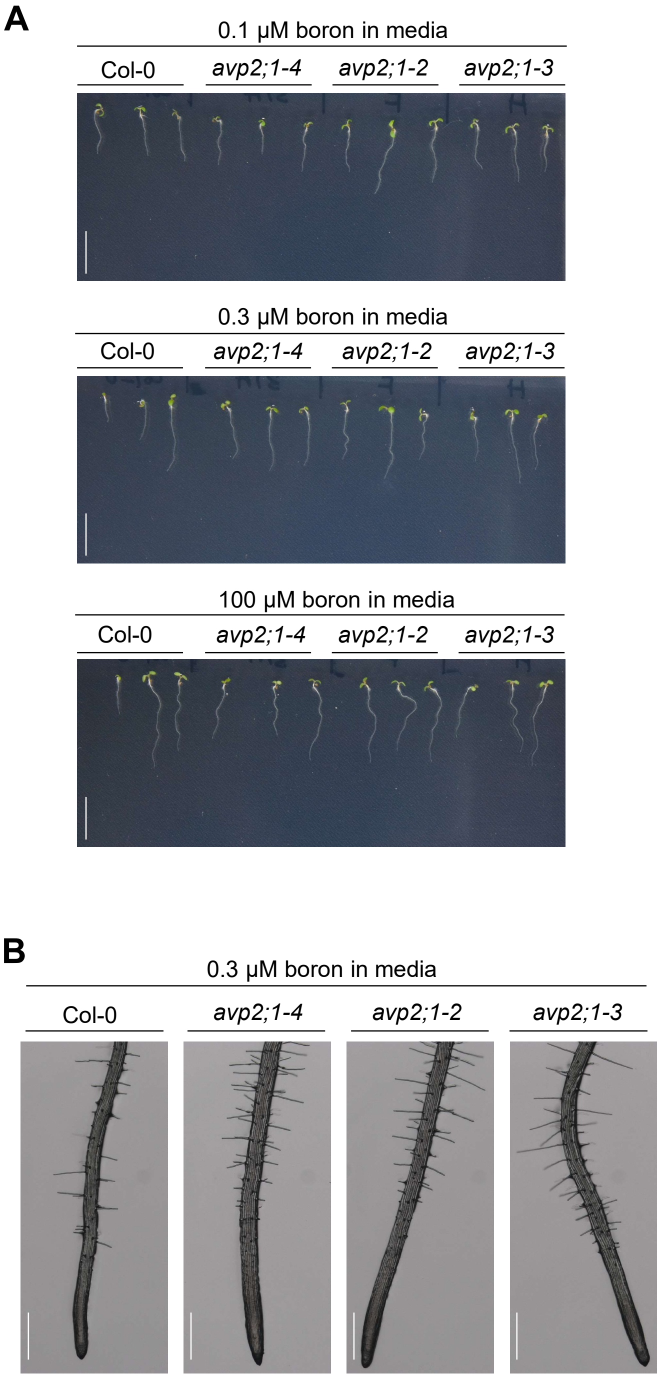

Supplementary Figure S2. Observation of the root cells of wildtype Col-0 and *avp2;1* mutants. **(A)** Images of wildtype Col-0 and *avp2;1* mutant lines grown under low (0.1 μM), mildly low (0.3 μM) and normal (100 μM) boron conditions in solid media for root cell observation. (Scale bar: 10 mm). **(B)** Stereomicroscopic images of wildtype Col-0 and *avp2;1* mutants root tips under mildly low (0.3 μM) boron conditions (scale bar: 400 μm). Plants were grown for 5 days for this analysis.

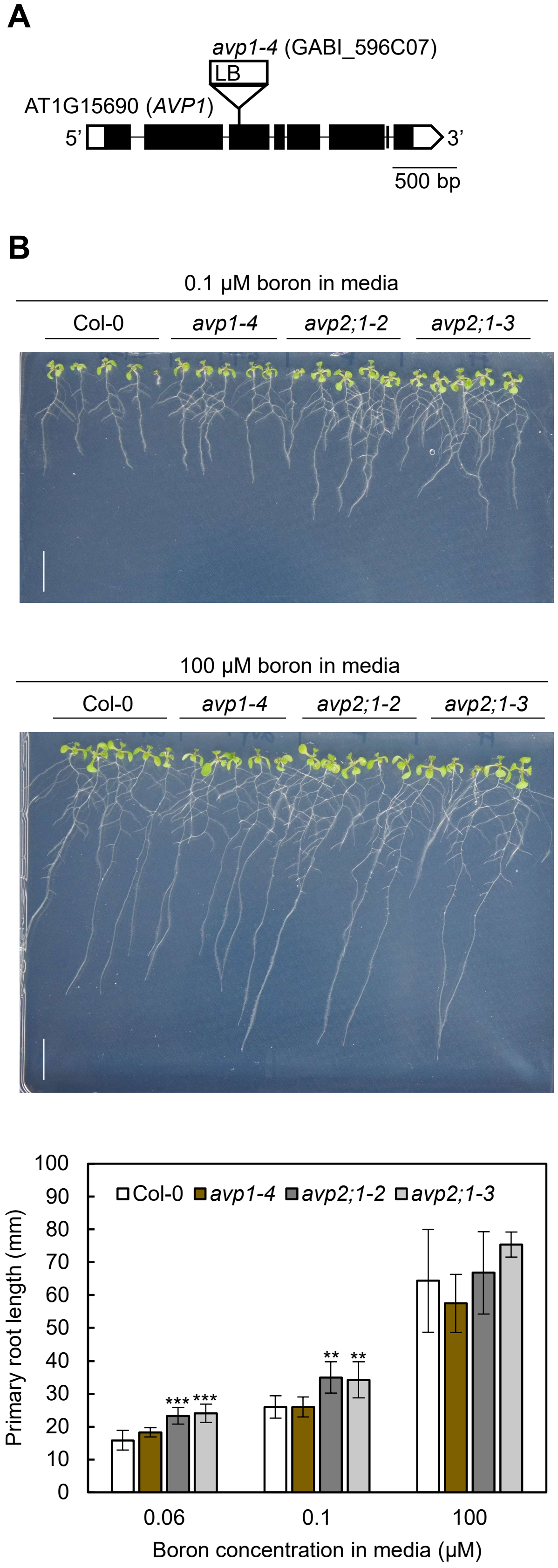

Supplementary Figure S3. Growth test of wildtype Col-0, *avp1* mutant line and *avp2;1* T-DNA lines under low and normal boron conditions. **(A)** Gene structure of *AVP1* (AT1G15690) showing insertion position of T-DNA. **(B)** Col-0, a*vp1-4, avp2;1-2* and *avp2;1-3* primary root length under low (0.1 µM) and normal (100 µM) boron conditions. (Scale bar: 10 mm). Plants were grown for 9 days. Mean ± SDs are shown (n=6-13). ***P < 0.001,**P < 0.01, compared with wildtype Col-0 under the same boron conditions (Dunnett's test)

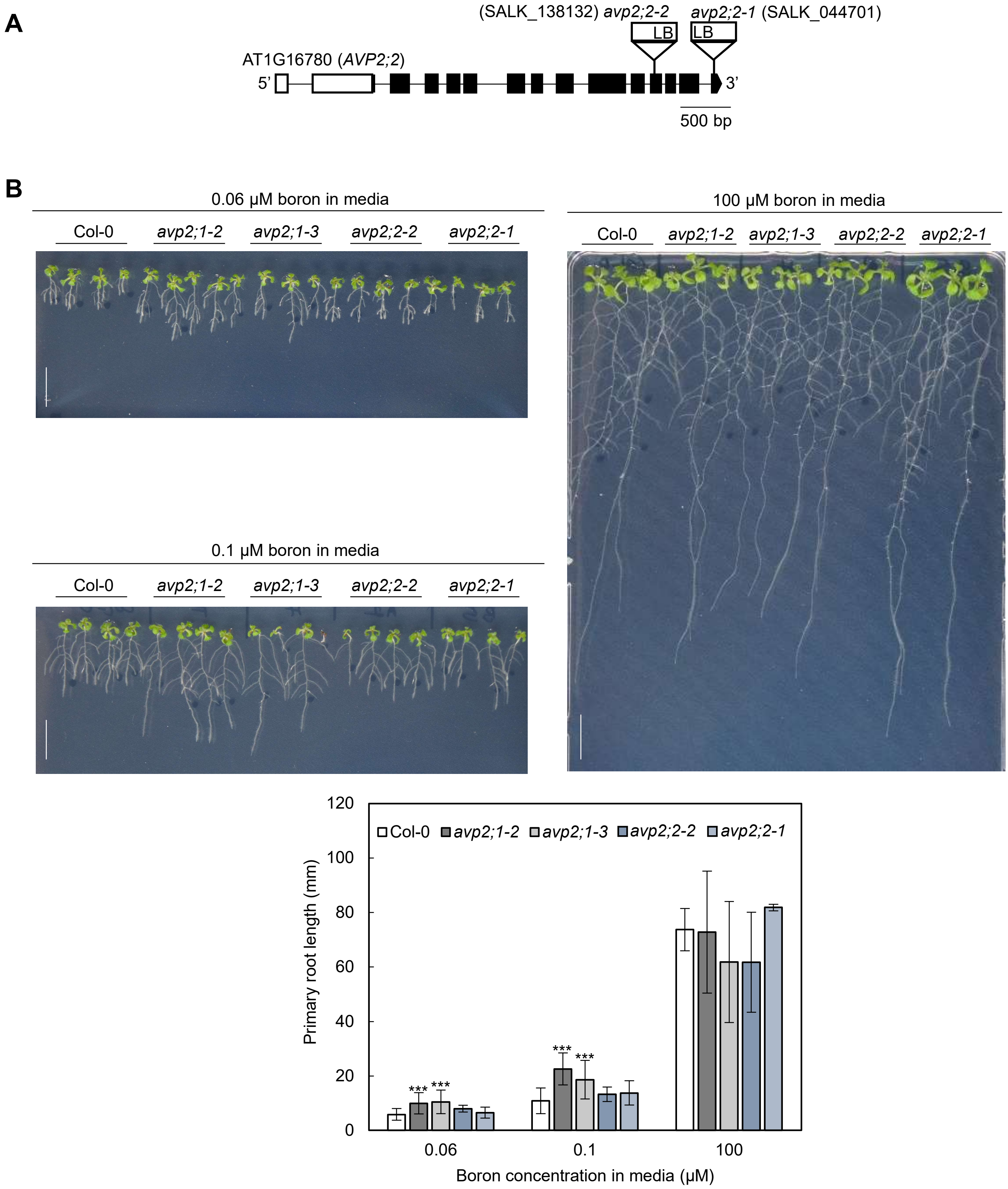

Supplementary Figure S4. Growth analysis of wildtype Col-0 and *avp2;2* mutants under various boron concentrations. **(A)** Gene structure of *AVP2;2* (AT1G16780) showing insertion positions of T-DNA. **(B)** Primary root length of wildtype Col-0, *avp2;1* and *avp2;2* mutants plant lines grown under very low (0.06 µM), low (0.1 µM) and normal (100 µM) boron conditions. (Scale bar=10 mm). *avp2;2-2* and *avp2;2-1* are SALK_138132 and SALK_044701 lines, respectively. Plants used for analysis are 11-d-old. Mean ±SDs are shown (n=15-27). ****P* < 0.001 compared with wildtype Col-0 under the same boron conditions (Tukey-Kramer test)
